# Synthesis of Chiral 1,4-Disubstituted-1,2,3-Triazole Derivatives from Amino Acids

**DOI:** 10.3390/molecules14125124

**Published:** 2009-12-09

**Authors:** Michael Klein, Karin Krainz, Itedale Namro Redwan, Peter Dinér, Morten Grøtli

**Affiliations:** Department of Chemistry, Medicinal Chemistry, University of Gothenburg, SE-412 96 Göteborg, Sweden

**Keywords:** [3+2] cycloaddition, azido alcohols, 1,4-disubstituted-1,2,3-triazoles

## Abstract

A versatile method for the synthesis of chiral 1,4-disubstituted-1,2,3-triazole derivatives starting from easily accessible naturally occurring D-or L-amino acids as chiral synthons is described. The amino acids were converted into azido alcohols, followed by copper catalyzed [3+2] cycloaddition reactions between the azido alcohols and methyl propiolate and subsequent ester aminolysis with primary and secondary amines furnished the target compounds, which were obtained in excellent yields with no racemization. Docking of selected target compounds shows that the chiral 1,4-disubstituted-1,2,3-triazoles derivatives has the potential of mimicking the binding mode of known purine analogues.

## 1. Introduction

1,2,3-Triazoles are an important class of heterocycles due to their wide range of applications as synthetic intermediates and pharmaceuticals [1,2]. Several therapeutically interesting 1,2,3-triazoles have been reported, including anti-HIV agents [3,4,5,6], antimicrobial compounds [7], β_3_-selective adrenergic receptor agonists [8], kinase inhibitors [9,10] and other enzyme inhibitors [11,12]. The 1,2,3-triazole moiety is also present in a number of drugs, for example, the β-lactam antibiotic tazobactam [13] and the cephalosporin cefatrizine [14]. 

**Figure 1:**
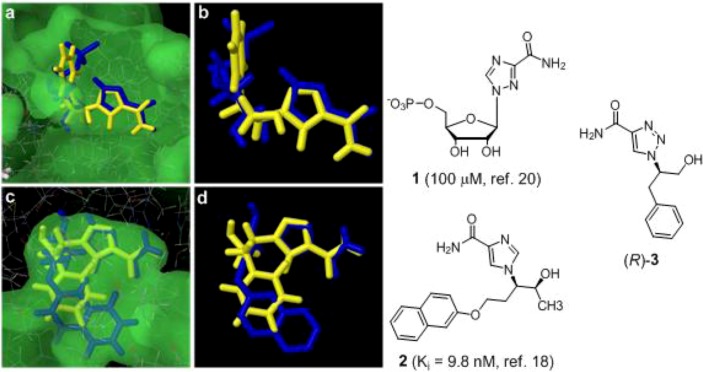
**(a)** Docked binding mode of (*R*)**-3** (yellow) compared to X-ray structure (1R6A) of ribavirin-5’-triphosphate (blue) 1 in the active site of the NS5MTase_DV_ in the dengue virus; (**b**) The docking shows that the NH2-groups of compound (R)-**3** and ribavirin-5’-monophosphate are almost superimposed and makes the same important interaction with the carbonyl functions of Leu17 and Leu20 in the protein; (**c**) Docked binding mode of (*R*)**-3** (yellow) compared to x-ray structure (1v7a) of inhibitor **2** in the active site of the ADA enzyme; (**d**) The imidazole part of compound (*R*)**-3** is almost superimposed over compound **2** and the phenyl ring points in (*R*)**-3** in the same direction as the naphtyl ring in **2**.

In our search for synthetic non-natural frameworks that could mimic nucleosides, we wanted to investigate the possibility of using 1,2,3-triazole moiety as a mimic of the imidazole part of the adenine system (Figure 1). Imidazole and triazole based nucleoside derivatives have been reported as potent enzyme inhibitors. Ribavarin [15], a 1,2,4-triazole based purine analogue, has high potency as an antiviral agent and is currently used in the clinic to inhibit RNA virus infections. Furthermore, imidazole based derivatives have been reported as potent adenosine deaminase (ADA) inhibitors (Figure 1) [16,17,18,19]. Therefore, we wanted to investigate the possibility of the 1,2,3-triazole scaffold to be docked into proteins that are inhibited of these nucleoside analogues and to develop efficient synthesis protocols for the preparation of 1,4-disubstituted-1,2,3-triazoles.

## 2. Results and Discussion

In order to explore the potential of the 1,4-disubstituted-1,2,3-triazoles as a possible scaffold for nucleoside inhibitors, compound (*R*)-**3** derived from D-phenylalanine, was docked into the nucleoside binding pocket of a viral enzyme of the Dengue virus (NS5MTase_DV_) [20] and adenosine deaminase [16,17,18,19]. Initially, the triazole scaffold was compared with the ribavirin nucleotide that inhibits the NS5MTase_DV_ enzyme. Upon entry into the cell, ribavirin gets converted to the 5’-triphosphate derivative before it binds to the enzyme (Figure 1A). However, due to poor electron density in the X-ray structure (1R6A) only the monophosphate has been modelled. Using Schrödinger’s Glide (XP docking mode), compound (*R*)-**3** docks into the same pose (yellow ligand) as the ribavirin 5’-monophosphate in the NS5MTase_DV_ enzyme suggesting that compound (*R*)-**3** mimics the binding mode of ribavirin (dark blue) (Figure 1B). The second example comes from the inhibition of the ADA enzyme. In this case, several crystal structures have been reported with imidazole-based nano-molar inhibitors [16,17,18,19]. 

Therefore, (*R*)-**3** was also docked into the ADA system in order to investigate the similarity of the 1,4-disubstituted-1,2,3-triazoles to the imidazole-based nucleoside analogue **2** (Figure 1C). The docking results from Glide (XP docking mode) show that our model compounds (yellow) superimposes the imidazole of the inhibitor in the binding pocket of the X-ray structure (Figure 1D). 

The docking results from NS5MTase_DV_ and adenosine deaminase (ADA) suggest that chiral 1,4-disubstituted-1,2,3-triazoles derivatives has the potential of mimicking the binding mode of known purine analogues.

**Figure 2:**
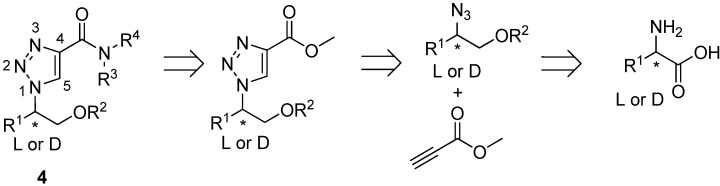
Retrosynthetic strategy for the generation of chiral 1,4-disubstituted-1,2,3-triazole derivatives.

The 1 and 4 position of the triazole scaffold allow diversification and the 1,4-disubstituted-1,2,3-triazoles should be easy accessible from [3+2] cycloaddition reactions between azides and alkynes using the copper catalyzed procedure reported by Meldal [21] and Sharpless [22] (Figure 2). We anticipated that by using azido alcohol derivatives, prepared from amino acids, a variety of different R-groups could be introduced resulting in chiral 1,4-disubstituted-1,2,3-triazoles. Furthermore, by using propiolate in the [3+2] cycloaddition reactions an ester functionality would be present in 4 position of the disubstituted-1,2,3-triazoles and allow further derivatisation, for example by reactions with primary or secondary amines.

Here, we report an efficient synthesis of compounds represented by structure **4** (Figure 2) by performing [3+2] cycloaddition reactions followed by ester aminolysis. According to the general strategy outlined in Figure 2, our synthesis of the 1,4-disubstituted-1,2,3-triazoles commenced with the preparation of amino alcohols **10-14** by reduction of the corresponding amino acids **5-9** (Table 1).

Both L-Phe and D-Phe were selected in order to obtain the two different stereoisomers of 1,4-disubstituted-1,2,3-triazoles containing an aromatic R-group. L-Homo-Phe was selected in order to obtain 1,4-disubstituted-1,2,3-triazoles with the phenyl group further away from the triazole ring. These aromatic groups could potentially make interactions with hydrophobic pockets adjacent to the nucleoside binding site in target proteins [23]. We also wanted to explore the use of amino acids with alkyl side chains and selected L-Val and L-Ser. Although the copper(I)-catalyzed 1,3-dipolar cycloaddition is insensitive to functional group interference, a benzyl ether derivative of L-Ser was used in order to facilitate column chromatography of the target compound. The amino acids were reduced using LiAlH_4_ and the resulting amino alcohols converted to the corresponding azides via treatment with TfN_3_. In this way, a variety of azides derived from various amino alcohols were obtained in good yields (70-76% yields) [24,25,26,27,28,29].

**Table 1:** Synthesis of azido alcohols. 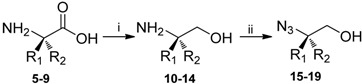

Compound	R^1^	R^2^	Yield (%)
**5**	H	CH_2_Ph	-
**6**	CH_2_Ph	H	-
**7**	H	CH_2_CH_2_Ph	-
**8**	H	CH(CH_3_)_2_	-
**9**	H	CH_2_OCH_2_Ph	-
**10**	H	CH_2_Ph	79
**11**	CH_2_Ph	H	91
**12**	H	CH_2_CH_2_Ph	92
**13**	H	CH(CH_3_)_2_	96
**14**	H	CH_2_OCH_2_Ph	97
**15**	H	CH_2_Ph	76
**16**	CH_2_Ph	H	72
**17**	H	CH_2_CH_2_Ph	74
**18**	H	CH(CH_3_)_2_	70
**19**	H	CH_2_OCH_2_Ph	73

(i) LiAlH_4_, THF, 16 h, reflux. (ii) TfN_3_, DMAP, DCM, 15 h, RT.

In general, the copper(I)-catalyzed 1,3-dipolar cycloaddition is known as a mild reaction proceeding well in aqueous solutions without protection from oxygen. A range of different reaction conditions have been reported, particularly with respect to generation of the active Cu^I^ species. Sources of Cu^I^ include Cu^I^ salts, most commonly copper iodide [21], *in situ* reduction of Cu^II^ salts, particularly Cu^II^ sulfate [22], and comproportionation of Cu^0^ and Cu^II^ [30,31]. Recent reports suggest that nitrogen-based ligands can stabilize the Cu^I^ oxidation state under aerobic, aqueous conditions and promote the desired transformation [32]. 

We got very good results using 5 mol % of sodium ascorbate and 1 mol % of copper(II) sulphate in a 1:1 mixture of water and *tert*-butyl alcohol (Table 2). The reaction between methylene propiolate and **15** or **18** using these reaction conditions furnished the two 1,4-disubstituted triazole products **24** and **25** in 71% and 74% isolated yields respectively, after stirring at RT in a capped vial. The reactions were monitored by TLC and showed complete conversion after 15 h. The product yields in the reactions therefore reflect the efficiency of product purification rather than the coupling efficiency.

**Table 2:** Synthesis of 1,4-disubstituted-1,2,3-triazoles. 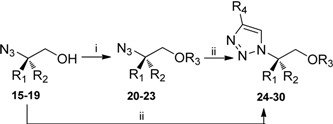

Compound	R^1^	R^2^	R^3^	R^4^	Yield (%)
**20**	H	CH_2_Ph	CH_2_Ph	-	87
**21**	CH_2_Ph	H	CH_2_Ph	-	91
**22**	H	CH_2_CH_2_Ph	CH_2_Ph	-	92
**23**	H	CH_2_OCH_2_Ph	Si(CH_3_)_2_C(CH_3_)_3_	-	67
**24**	H	CH_2_Ph	H	CO_2_CH_3_	71
**25**	H	CH(CH_3_)_2_	H	CO_2_CH_3_	74
**26**	H	CH_2_Ph	H	Ph	97
**27**	H	CH_2_Ph	CH_2_Ph	CO_2_CH_3_	83
**28**	CH_2_Ph	H	CH_2_Ph	CO_2_CH_3_	90
**29**	H	CH_2_CH_2_Ph	CH_2_Ph	CO_2_CH_3_	82

(i) BnCl, NaH, TBAI, THF, 15 h, RT; or TBDMSiCl, imidazole, DMF, 15 h, RT; (ii) sodium ascorbate, CuSO_4_ in H_2_O:*t*-BuOH (1:1 v/v), 15h, RT.

In order to improve the efficiency of purification of the products by column chromatography we decided to block the hydroxyl function of the azido alcohols either as benzyl ethers (as in compounds **20**-**22**) or as TBDMS ethers (as in compound **23**). In addition, the benzyl ethers could make potential interactions in the phosphate-and sugar binding area of the ATP binding site of target kinases [23]. 

The protected azido derivatives **20**-**22** were only partially soluble in water: *t-*BuOH (1:1 v/v) and the cycloaddition reactions with methylene propiolate were attempted in water: THF (1:2 v/v) instead. The corresponding 1,4-disubstituted triazole products **26-28** were obtained high yields (83–97% yields). However, the reaction between methylene propiolate and the TBDMS-protected azido alcohol did not proceed to completion, and the corresponding 1,4-disubstituted triazole **30** was only obtained in 66% yield. Prolonged reaction times (30 h) or running the reaction at elevated temperature (50 ^o^C for 15 h) did not improve the yield (data not shown). Steric interference of the bulky TBDMS ether in the cycloaddition reaction is a possible explanation for this effect. 

**Scheme 1:**
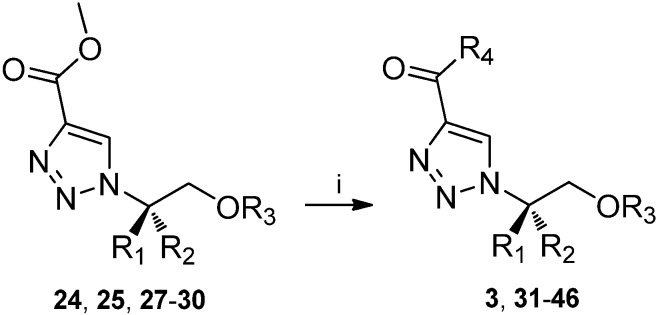
Derivatisation of ester functionality with different amides. i. Amines, NaOMe, MeOH, 15 h, RT.

As well as high yields and prevention of by-product formation, it is essential to avoid racemization when functionalizing enantiomerically pure compounds. Hence, a chiral HPLC system was used to analyze the [3+2] cycloaddition products **27** and **28**. The two enantiomers separated well on the chiral HPLC column with retention times of 16.6 min (**27**) and 13.4 min (**28**) respectively. No racemization could be detected. (Scheme 1)

The ester functionality of the disubstituted-1,2,3-triazoles was further derivatised by reaction with primary and secondary amines. Compound **24** and **25** were reacted with 7N NH_3_ in MeOH for 15 h at RT and the corresponding target compounds were obtained in 97% and 93% isolated yields respectively. However, reacting **24** with other amines (3 eq) such as butylamine, benzylamine, diethylamine, morpholine or ethanolamine in MeOH, resulted in sluggish reactions. Prolonged reaction times (30 h), larger excess of amines (10 eq) or elevated temperature, either by classical thermal heating (50 ^o^C, 10 h) or by microwave assisted heating (100 ^o^C, 10-60 min in a sealed vessel) had only a marginal effect on the yield.

The reactions were repeated in the presence of 2 eq of sodium methoxide and TLC showed complete conversion after 15 h at RT. Nevertheless, several products were formed and the target compounds **31**-**35** were only isolated in low to moderate yields (4-58%). In general, the primary amines gave the best result while the secondary amines proceeded more slowly (Table 3). 

**Table 3:** Synthesis of 4-carbamoyl-1,2,3-triazoles.

Compound	R^1^	R^2^	R^3^	R^4^	Yield (%)
**(*S*)-3**	H	CH_2_Ph	H	NH_2_	97
**31**	H	CH_2_Ph	H	NH(CH_2_)_3_CH_3_	58
**32**	H	CH_2_Ph	H	NH(CH_2_)_2_OH	4
**33**	H	CH_2_Ph	H	NHCH_2_Ph	35
**34**	H	CH_2_Ph	H	N(CH_2_CH_3_)_2_	12
**35**	H	CH_2_Ph	H	N(CH_2_CH_2_)_2_O	30
**36**	H	CH(CH_3_)_2_	H	NH_2_	35
**37**	H	CH_2_Ph	CH_2_Ph	NH_2_	92
**38**	H	CH_2_Ph	CH_2_Ph	NH(CH_2_)_3_CH_3_	93
**39**	H	CH_2_Ph	CH_2_Ph	NH(CH_2_)_2_OH	81
**40**	H	CH_2_Ph	CH_2_Ph	NHCH_2_Ph	87
**41**	H	CH_2_Ph	CH_2_Ph	N(CH_2_CH_3_)_2_	8
**42**	H	CH_2_Ph	CH_2_Ph	N(CH_2_CH_2_)_2_O	68
**43**	CH_2_Ph	H	CH_2_Ph	NH(CH_2_)_3_CH_3_	98
**44**	H	CH_2_OCH_2_Ph	CH_2_Ph	NH_2_	88
**45**	H	CH_2_OCH_2_Ph	CH_2_Ph	NH(CH_2_)_3_CH_3_	93
**46**	H	CH_2_OCH_2_Ph	Si(CH_3_)_2_C(CH_3_)_3_	NH_2_	92

On the other hand, when the 4-carboxy methyl ester-triazole **27** was reacted with 7N NH_3_ in MeOH or amines in the presence of sodium methoxide the 4-carbamoyltriazoles **37-42** were obtained in high yields. Ammonia and primary amines gave excellent yields (81–92%) while diethyl amine and morpholine did not go to completion and gave the corresponding 4-carbamoyltriazoles in 8 and 68% yields respectively. The 4-carbamoyltriazoles **43-46** were also obtained in excellent yields (88–98%). In all cases TLC showed clean reactions with only formation of target compounds. The results clearly show that it is essential to block the primary alcohol in order to prevent side-reaction and to secure high yields of the 4-carbamoyltriazoles. 

The two enantiomers **38** and **43** were analyzed by chiral HPLC to determine if any racemization occurred in the amination step. The two enantiomers separated well on the chiral HPLC column with retention times of 21.2 min (38) and 14.9 min (43) respectively. No racemization could be detected.

## 3. Experimental

### 3.1. General

(*S*)-2-Amino-3-phenylpropionic acid, (*S*)-2-amino-3-methylbutyric acid, (*R*)-2-amino-3-phenyl-propionic acid, (*S*)-2-amino-4-phenylbutyric acid and (*S*)-2-amino-3-benzyloxypropionic acid (**40**) were obtained from Sigma Aldrich and Bachem. Trifluoromethanesulfonic anhydride was obtained from Acros. All other chemicals were reagent grade or better obtained from reputable suppliers. ^1^H- and ^13^C-NMR spectra were recorded on a JEOL JNM-EX 400 spectrometer at 400 and 100 MHz, respectively. Optical rotation was measured with a Perkin Elmer Polarimeter 341 LC. The reactions were monitored by TLC, on silica plated aluminium sheets (Silica gel 60F254, E. Merck) in case of flash chromatography, Merck silica gel CC (230-400 mesh) was used. Elemental analyses were performed at H Kolbe Mikroanalytisches Laborium, Mühlheim an der Ruhr, Germany. 

Chiral HPLC analysis was performed on a Varian system (9012 pump, flow 1 mL min^−1^; 9050 detector at 254 nm) using a Chiralpac AD column (250 × 4.6 mm) and isocratic elution with 10% *i*-PrOH/*n*-hexane (method A) or 5% *i*-PrOH/*n*-hexane (method B).

### 3.2. General procedure for preparation of amino alcohols

A solution of lithium aluminium hydride (1.4 eq) in dry THF (2 mL/mmol amino acid) was stirred and cooled in an ice bath. The amino acid (1 eq) was added and the mixture was stirred at 0 ^o^C for 1 h, warmed to room temperature, and stirred for an additional 1 h. The reaction mixture was then heated at reflux for 16 h. The solution was cooled in an ice bath and diluted slowly with ether (30 mL). Water (7.5 mL) and 15% NaOH (2.5 mL) were added dropwise to the solution in succession. The mixture was filtered through Celite to remove the aluminium salts. The Celite was washed with ether (2 × 15 mL). The filtrate was collected and the solvent was removed under reduced pressure affording the amino alcohol. The amino alcohols were used in the following steps without further purification.

*(S)-2-Amino-3-phenylpropan-1-ol* (**10**)*:* The target compound was prepared from **5** (24.2 mmol, 4 g) using the general procedure described above and obtained as a yellow solid (2.9 g, 79% yield). Analytical data was consistent with the literature [33].

*(R)-2-Amino-3-phenylpropan-1-ol* (**11**)*:* The target compound was prepared from 6 (18.2 mmol, 3.00 g) using the general procedure described above and obtained as a yellow oil (2.49 g, 91% yield). The amino alcohol was used for the following steps without further purification [34].

*(S)-2-Amino-4-phenylbutan-1-ol* (**12**)*:* The target compound was prepared from 7 (5.5 mmol, 1.00 g) using the general procedure described above and obtained as a yellow oil (50 mg, 92% yield). Analytical data was consistent with the literature [34].

*(S)-2-Amino-3-methylbutan-1-ol* (**13**)*:* The target compound was prepared from 8 (8.23 mmol, 965 mg) using the general procedure described above and obtained as a yellow oil (805 mg, 96% yield). Analytical data was consistent with the literature [35].

*(R)-2-Amino-3-benzyloxypropan-1-ol* (**14**)*:* The target compound was prepared from 9 (10.24 mmol, 2.00 g) using the general procedure described above and obtained as an oil (1.80 g, 97% yield). The amino alcohol was used for the following steps without further purification. ^1^H-NMR (CDCl_3_) δ 3.03–3.10 (1H, m), 3.37–3.62 (2H, m), 3.43–3.50 (2H, m), 4.49 (2H, s), 7.24–7.37(5H, m). Analytical data was consistent with the literature [35].

### 3.3. General procedure for preparation of azido alcohols by diazo transfer

To a solution of amino alcohol (1 eq) and DMAP (2.5 eq) in CH_2_Cl_2_ (1mL/mmol amino alcohol) triflyl azide solution (4.4 eq) was added dropwise with stirring and exclusion of moisture. The reaction mixture was stirred at rt for 15 h. The reaction mixture was concentrated under reduced pressure and purified by flash chromatography.

*(S)-2-Azido-3-phenylpropan-1-ol* (**15**): The target compound was prepared from **10** (19.27 mmol, 2.91 g) using the general procedure described above and purified by flash chromatography on silica gel using EtOAc/hexane(1:2 v/v) as eluent. The target compound was obtained as a yellow oil (2.45 g, 66% yield). Analytical data was consistent with the literature [28].

*(R)-2-Azido-3-phenylpropan-1-ol* (**16**): The target compound was prepared from **11** (16.4 mmol, 2.49 g) using the general procedure described above and purified by flash chromatography on silica gel using EtOAc/hexane (1:2 v/v) as eluent. The target compound was obtained as a yellow oil (2.10 g, 72% yield). ^1^H-NMR (CDCl_3_) δ 2.76–3.14 (2H, m), 3.55–3.69 (2H, m), 3.77–3.85 (1H, m), 7.20–7.35 (5H, m); ^13^C-NMR (CDCl_3_) δ 36.79 (-), 62.9 (+), 70.51 (-), 127.43 (+), 128.59 (+), 128.91 (+), 141.05 (q); Anal. Calc. for C_8_H_11_N_3_O: C, 64.39; H, 7.45; N, 28.17. Found: C, 64.44; H, 7.46; N, 28.28.

*(S)-2-Azido-4-phenylbutan-1-ol* (**17**): The target compound was prepared from **12** (5.03 mmol, 830 mg) using the general procedure described above and purified by flash chromatography on silica gel using EtOAc/hexane (1:2 v/v) as eluent. The target compound was obtained as a clear oil (710 mg, 74% yield). ^1^H-NMR (CDCl_3_) δ 1.78–1.85 (2H, m), 2.67–2.85 (2H, m), 3.42–3.49 (1H, m), 3.55–3.73 (2H, m), 3.68–3.72 (2H, m), 7.18–7.34 (5H, m); ^13^C-NMR (CDCl_3_) δ 32.3 (-), 32.4 (-), 63.5 (+), 65.5 (-), 126.4 (+), 128.6 (+), 128.8 (+), 141.0 (q); Anal. Calc. for C_10_H_13_N_3_O: C, 62.81; H, 6.85; N, 21.97. Found: C, 62.69; H, 6.79; N, 21.88.

*(S)-2-Azido-3-methylbutan-1-ol* (**18**): The target compound was prepared from **13** (7.8 mmol, 805 mg) using the general procedure described above and purified by flash chromatography on silica gel using EtOAc/hexane (1:3 v/v) as eluent, affording the target compound as a yellow oil (698 mg, 70% yield). Analytical data was consistent with the literature [35].

*(R)-2-Azido-3-benzyloxypropan-1-ol* (**19**): The target compound was prepared from **14** (10.7 mmol, 1.8 g) using the general procedure described above and purified by flash chromatography on silica gel using EtOAc/hexane (1:2 v/v) as eluent. The target compound was obtained as an oil (1.51 g, 73% yield). Analytical data was consistent with the literature [36].

### 3.4. General procedure for benzylation of the hydroxyl functionality

The azido alcohol (1 eq) was dissolved in dry THF (10 mL), NaH (suspension in mineral oil 60%, 2 eq) was added and the suspension was stirred 5 min at rt. Benzyl bromide (2 eq) and TBAI (0.05 eq) were added and the reaction mixture stirred for 15 h. The reaction was quenched with sat. NH_4_Cl aq (15 mL) at which the reaction mixture became clear, and was then extracted with EtOAc (2 × 30 mL). The organic phases were pooled and washed once with brine, dried with MgSO_4_, filtered and concentrated under reduced pressure. The residue was purified by column chromatography on silica gel using hexane and hexane/EtOAc (95:5 v/v) as eluents.

*(S)-2-Azido-1-benzyloxy-3-phenylpropane* (**20**): The target compound was prepared from **15** (12.18 mmol, 2.16 g) using the general procedure described above and obtained as a yellow oil (2.8 g, 87% yield). ^1^H-NMR (CDCl_3_) δ 2.83–2.99 (2 H, m), 3.54–3.59 (2H, m), 3.63–3.68 (1H, m), 4.63 (2H, m), 7.24–7.45 (10 H, m); ^13^C-NMR (CDCl_3_) δ 37.30 (-), 62.81 (+), 71.91 (-), 73.46 (-), 126.90 (+), 127.73 (+), 127.89 (+), 128.52 (+), 128.68 (+), 129.38 (+), 137.43 (q), 137.91 (q); Anal. Calc. for C_16_H_17_N_3_O: C,71.87; H, 6.42; N, 15.72. Found: C, 71.90; H, 6.44; N, 15.75.

*(R)-2-Azido-1-benzyloxy-3-phenylpropane* (**21**): The target compound was prepared from **16** (5.64 mmol, 1.00 mg) using the general procedure described above and was obtained as a yellow oil (1.37 g, 91% yield). ^1^H-NMR (CDCl_3_) δ 2.82–3.01 (2 H, m), 3.54–3.62 (2H, m), 3.79–3.88 (1H, m), 4.65–4.72 (2H, m), 7.29–7.48 (10 H, m); ^13^C-NMR (CDCl_3_) δ 37.24 (-), 62.75 (+), 71.86 (-), 73.39 (-), 126.84 (+), 127.68 (+), 127.84 (+), 128.53 (+), 128.63 (+), 129.34 (+), 137.39 (q), 137.88 (q); Anal. Calc. For C_16_H_19_N_3_O: C, 72.57; H, 6.81; N, 14.93. Found: C, 71.67; H, 6.72; N, 15.04.

*(S)-2-Azido-1-benzyloxy-4-phenylbutane* (**22**): The target compound was prepared from **17** (3.32 mmol, 635 mg) using the general procedure described above and was obtained as a yellow oil (861 mg, 92% yield). ^1^H-NMR (CDCl_3_) δ 1.82–1.92 (2H, m), 2.68–2.91 (2H, m), 3.53–3.68 (3H, m), 4.62 (2H, s), 7.23–7.48 (10 H, m); ^13^C-NMR (CDCl_3_) δ 32.27 (-), 32.63 (-), 61.08 (+), 72.96 (-), 73.44 (-), 126.25 (+), 127.6 (+), 127.8 (+), 128.5 (+), 128.6 (+), 128.8 (+), 137.9 (q), 141.0 (q), Anal. Calc. for C_17_H_19_N_3_O: C, 72.57; H, 6.81; N, 14.93. Found: C, 72.59; H, 6.77; N, 15.00.

*((S)-2-Azido-3-benzyloxypropoxy)-tert-butyl-dimethylsilane* (**23**): Compound **19** (2.59 mmol, 538 mg) was dissolved in DMF (5 mL), TBDMS-Cl (3.38 mmol, 506 mg) and imidazole (6.5 mmol, 441 mg) were added and the mixture stirred 15 h at rt. The reaction mixture was concentrated under reduced pressure, extracted with EtOAc and NH_4_Cl_aq_ and purified by column chromatography on silica gel using hexane/EtOAc (99:1 v/v) as eluent. The target compound was obtained as an oil (775 mg, 93% yield). ^1^H-NMR (CDCl_3_) δ 0.13 (6H, s), 0.95 (9H, s), 3.54–3.85 (5H, m), 4.60 (2H, s), 7.29–7.43 (5H, m); ^13^C-NMR (CDCl_3_) δ -5.39 (*+*), 18.35 (q), 25.93 (*+*), 62.50 (N_3_*CH*CH_2_), 63.40 (+), 69.37 (-), 73.59 (-), 127.80 (+), 127.90 (+), 128.59 (+), 137.93 (q); Anal. Calc. for C_16_H_27_N_3_O_2_Si: C, 59.78; H, 8.47; N, 13.07. Found: C, 59.74; H, 8.42; N, 13.02.

### 3.5. General procedure for copper catalyzed cycloaddition reactions

The unprotected azido alcohol (1eq) and the alkyne (1.5 eq) were suspended in a 1:1 mixture of water and *tert-*butyl alcohol (5 mL/mmol azido alcohol). Benzyl protected azido alcohol (1 eq) and the alkyne (1.5 eq) were suspended in H_2_O/THF (1:2, v/v). Sodium ascorbate (0.1 eq as a 1 M solution in water) was added, followed by copper(II) sulfate (0.01 eq as a 0.1 M solution in water). The reaction mixture was stirred at rt for 15 h. The solvents were removed under reduced pressure and the residue adsorbed on silica gel and purified by column chromatography.

*1-((S)-1-Hydroxymethyl-2-phenethyl)-1H-1,2,3-triazole-4-carboxylic acid methyl ester* (**24**): The target compound was prepared from **15** (14.9 mmol, 2.64 g) and methyl propiolate (22.35 mmol, 1.87 g) using the general procedure described above. The product was purified by column chromatography on silica gel (2:1 EtOAc/hexane) affording the target compound as a pale oil (3.15 g, 81% yield). ^1^H- NMR (CDCl_3_) δ 3.28 (2H, d, *J =* 7.7 Hz), 3.89 (3H, s), 4.04–4.17 (2H, m), 4.75–4.81 (1H, m), 7.05–7.27 (5H m), 7.95 (1H, s); ^13^C-NMR (CDCl_3_) δ 37.35 (-), 52.15 (+), 63.50 (+), 65.56 (+), 127.25 (+), 128.46 (q), 129.10 (2x+), 135.89 (q), 139.06 (q), 161.03 (q); Anal. Calc. for C_13_H_15_N_3_O_3_: C, 59.76; H, 5.79; N, 16.08. Found: C, 59.87; H, 5.74; N, 15.93.

*1-((S)-1-Hydroxymethyl-2-methylpropyl)-1H-1,2,3-triazole-4-carboxylic acid methyl ester (**25**):* The target compound was prepared from **18** (5.43 mmol, 700 mg) and methyl propiolate (8.14 mmol, 684 mg) using the general procedure described above. The product was purified by column chromatography on silica gel (2:1 EtOAc/hexane) affording the target compound as a pale oil (984 mg, 85% yield). ^1^H-NMR (CDCl_3_) δ 0.76 (3H, d, 6.6 Hz), 1.08 (3H, d, 6.6 Hz), 2.30–2.43 (1H, m), 3.92 (3H, s), 4.00–4.07 (1H, m), 4.17–4.25 (1H, m), 8.17 (1H, s); ^13^C-NMR (CDCl_3_) δ 19.33 (+), 19.61 (+), 29.73 (+), 52.31 (+), 62.35 (-), 70.24 (+), 128.15 (+), 139.73 (q), 161.31 (q); Anal. Calc. for C_9_H_15_N_3_O_3_: C, 50.69; H, 7.09; N, 19.71; Found: C, 50.90; H, 6.92; N, 19.84.

*1-((S)-(1-Hydroxymethyl-2-phenethyl)-4-phenyl-1H,1,2,3-triazole* (**26**): The target compound was prepared from **15** (2 mmol, 365 mg) and phenylacetylene (2.5 mmol, 275 μL) using the general procedure described above. The crude product was purified by column chromatography on silica gel (2:3 EtOAc/hexane) affording the desired product as a colourless solid (512 mg, 98% yield). ^1^H-NMR (CDCl_3_) δ 3.22–3.35 (2H, m), 4.05–4.25 (2H, m), 4.67–4.73 (1H, m), 7.07–7.12 (2H, m), 7.19–7.39 (6H, m), 7.50 (1H, s), 7.61–7.67 (2H, m); ^13^C-NMR (CDCl_3_) δ 37.97(+), 64.19 (-), 65.55 (+), 120.70 (+), 125.75 (+), 127.30 (+), 128.30 (+), 128.96 (+), 129.00 (+), 129.21 (+), 130.46 (q), 135.76 (q), 136.77 (q); Anal. Calc. for C_17_H_17_N_3_O: C, 73.10; H, 6.13; N, 15.04. Found: C, 72.94; H, 6.09; N, 14.98.

*1-((S)-1-Benzyloxymethyl-2-phenethyl)-1H-1,2,3-triazole-4-carboxylic acid methyl ester* (**27**): The target compound was prepared from **20** (5.6 mmol, 1.50 g) and methyl propiolate (8.4 mmol, 711 mg) using the general procedure described above. The crude product was obtained as a pale oil (1.63 g, 83% yield). ^1^H-NMR (CDCl_3_) δ 3.21–3.32 (2H, m), 3.75–3.84 (2H, m), 3.92 (3H, s), 4.41–4.51 (2H, m), 4.84–4.98 (1H, m), 7.01–7.05 (2H, m), 7.16–7.36 (8H, m), 8.08 (1H, s); ^13^C-NMR (CDCl_3_) δ 37.81 (-), 52.30 (+), 63.59 (+), 70.13 (-), 73.66 (-), 127.38 (+), 127.96 (+), 128.23 (+), 128.71 (+), 128.96 (+), 129.04 (+), 135.94 (q), 137.19 (q), 139.51 (+), 161.31 (q); Anal. Calc. for: C_20_H_21_N_3_O_3_: C, 68.36; H, 6.02; N, 13.66. Found: C, 68.29; H, 5.98; N, 13.55; Optical rotation: [α]^20^_D_= +53,33 (c 1,06 in CHCl_3_); *t*_R_ (method A): 16.3.

*1-((R)-1-Benzyloxymethyl-2-phenethyl)-1H-1,2,3-triazole-4-carboxylic acid methyl ester* (**28**): The target compound was prepared from **21** (3.7 mmol, 1.00 g) and methyl propiolate (5.6 mmol, 474 mg) using the general procedure described above. The crude product was obtained as a yellow oil (1.18 mg, 90% yield). ^1^H-NMR (CDCl_3_) δ 3.21–3.33 (2H, m), 3.75–3.84 (2H, m), 3.93 (3H, s), 4.41–4.52 (2H, m), 4.89–4.97 (1H, m), 7.01–7.06 (2H, m), 7.17–7.37 (8H, m), 8.08 (1H, s); ^13^C-NMR (CDCl_3_) δ 37.85 (-), 52.30 (+), 63.56 (+), 70.16 (-), 73.68 (-), 127.39 (+), 127.98 (+), 128.24 (Ph), 128.73 (Ph), 128.98 (Ph), 129.06 (Ph), 135.98 (q), 137.21 (q), 139.47 (+), 161.40 (q); Anal. Calc. for C_20_H_21_N_3_O_3_: C, 68.36; H, 6.02; N, 11.96. Found: C, 68.30; H, 5.99; N, 11.87; Optical rotation: [α]^20^_D_= -50,94 (c 1,02 in CHCl_3_); *t*_R_ (methode A): 13.4.

*1-((S)-1-Benzyloxymethyl-3-phenylpropyl)-1H-1,2,3-triazole-4-carboxylic acid methyl ester* (**29**): The target compound was prepared from **22** (1.49 mmol, 400 mg) and methyl propiolate (2.14 mmol, 180 mg) using the general procedure described above. The crude product was obtained as a yellow oil (473 mg, 88% yield). ^1^H-NMR (CDCl_3_) δ 1.85–2.02 (2H, m), 2.57–2.71 (2H, m), 3.44–3.55 (2H, m), 3.84 (3H, s), 4.18–4.28 (2H, m), 4.46–4.59 (1H, m), 7.13–7.19 (2H, m), 7.23–7.37 (8H, m), 7.82 (1H, s); ^13^C-NMR (CDCl_3_) δ 32.62 (-), 33.98 (-), 49.67 (+), 52.54 (+), 71.75 (-), 73.38 (-), 126.06 (+), 127.86 (+), 128.58 (+), 138.36 (q), 139,81 (q), 141.84 (q), 161.38 (q); Anal. Calc. for C_21_H_23_N_3_O_3_: C, 68.82; H, 6.34; N, 11.74. Found: C, 69.03; H, 6.39; N, 11.85.

*1-[(S)-1-Benzyloxymethyl-2-(tert-butyl-dimethylsilanyloxy)-ethyl]-1H-1,2,3-triazole-4-carboxylic acid methyl ester* (**30**)**:** The target compound was prepared from **23** (1.21 mmol, 390 mg) and methyl propiolate (1.82 mmol, 153 mg) using the general procedure described above. The crude product was purified by column chromatography on silica gel (1:3 EtOAc/hexane) affording the product as an oil (382 mg, 82% yield). ^1^H-NMR (CDCl_3_) δ -0.11(3H, s), -0.10 (3H, s), 0.74 (9H, s), 3.77–3.88 (5H, m), 3.89–4.04 (2H, m), 4.37–4.46 (2H, m), 4.79–4.87 (1H, m), 7.11–7.25 (5H, m), 8.16 (1H, s); ^13^C-NMR (CDCl_3_) δ -5.88 (*+*), -5.85 (+), 17.87 (Q), 25.50 (*+*), 51.80 (+), 61.99 (-), 62.64 (+), 67.74 (-), 73.24 (-), 125.56 (+), 127.79 (+), 128.31 (+), 137.01 (q), 139.15 (q), 161.03 (q); Anal. Calc. for C_20_H_31_N_3_O_4_Si: C, 59.23; H, 7.70; N, 10.36. Found: C, 58.99; H, 7.79; N, 10.28.

### 3.6. General procedure for ester-amide conversion

Reaction with ammonia: The ester was dissolved in 7N NH_3_ in MeOH (1.5 mL) and stirred at rt overnight. The solvent was removed under reduced pressure and the desired product was purified by recrystallization or by column chromatography on silica gel.

Reaction with all other amines: Sodium (2 eq) was dissolved in MeOH (3 mL) under nitrogen followed by addition of amine (3 eq). A solution of the ester (1 eq) dissolved in MeOH (0.5 mL) was added to the reaction mixture. The reaction was stirred under nitrogen at rt for 15 h. The reaction was quenched with 1M HCl aq (1 mL) and extracted with CHCl_3_ (2 x 20 mL). The organic layer was washed with brine, separated, dried with MgSO_4_. The solvent was removed under reduced pressure and the crude product purified by column chromatography on silica gel or by recrystallization.

*1-((S)-1-Benzyl-2-hydroxyethyl)-1H-1,2,3-triazole-4-carboxylic acid amide* (**(S)-3**): The target compound was obtained by reacting **24** (0.89 mmol, 233 mg) with NH_3_ following the procedure above. The desired product was recrystallized from EtOH affording 3 as a white solid (184 mg, 97% yield). ^1^H- NMR (DMSO) δ 3.13–3.26 (2H, m), 3.75–3.84 (2H, m), 4.86–4.93 (1H, m), 5.16 (1H, t), 7.09–7.23 (5H, m), 7.40 (1H, s), 7.77 (1H, s), 8.53 (1H, s); ^13^C-NMR (DMSO) δ 36.53 (-), 63.10 (-), 64.62 (+), 125.96 (+), 126.49 (*+*), 128.43 (*+*), 128.80 (*+*), 137.12 (q), 142.46 (q), 161.65 (q); Anal. Calc. for: C_12_H_14_N_4_O_2_: C, 58.53; H, 5.73; N, 22.75. Found: C, 58.39; H, 5.64; N, 22.78.

*1-((S)-1-Benzyl-2-hydroxyethyl)-1H-1,2,3-triazole-4-carboxylic acid butylamide* (**31**): The target compound was obtained by reacting **24** (0.76 mmol, 200 mg) with butyl amine (2.38 mmol, 230 μL) following the procedure described above. The crude product was purified by column chromatography on silica gel (2:3 EtOAc/hexane, then 2:1 EtOAc/hexane + 10% MeOH) affording compound **31** as a white solid (100 mg, 58% yield). ^1^H-NMR (CDCl_3_) δ 0.87–0.95 (3H, m), 1.30–1.61 (4H, m), 3.19–3.28 (2H, m), 3.37–3.42 (2H, m), 3.98–4.05 (2H, m), 4.77–4.84 (1H, m), 7.06–7.10 (2H, m), 7,14 (1H, t), 7.18–7.28 (3H, m), 8.07 (1H, s); ^13^C-NMR (CDCl_3_) δ 13.95 (*+*), 20.29 (-), 31.71 (-), 37.73 (-), 39.12 (-), 63.61(-), 65.46 (+), 126.00 (+), 127.43 (*+*), 129.05 (*+*), 129.13 (*+*), 136.27 (q), 142.19 (q), 160.48 (q); Anal. Calc. for: C_16_H_22_N_4_O_2_: C, 63.56; H, 7.33; N, 18.53. Found: C, 63.51; H, 7.23; N, 18.41.

*1-((S)-1-Benzyl-2-hydroxyethyl)-1H-1,2,3-triazole-4-carboxylic acid (2-hydroxy-ethyl)-amide* (**32**): The target compound was obtained by reacting **24** (0.55 mmol, 145 mg) with ethanol amine (1.6 mmol, 100 μL) following the procedure described above. The crude product was purified by column chromatography on silica gel using CHCl_3_/MeOH (9:1 v/v) as eluent. The title compound was obtained as an oil (7 mg, 4% yield). ^1^H-NMR (CD_3_OD) δ 3.18–3.36 (2H, m), 3.46–3.52 (2H, m), 3.66–3.71 (2H, m), 3.91–4.04 (2H, m), 4.64–4.69 (1H, m), 7.07–7.29 (5H, m), 8.27 (1H, s); ^13^C-NMR (CDCl_3_) δ 38.14 (-), 42.46 (-), 61.37 (-), 64.40 (-), 66.84 (+), 126.99 (+), 127.73 (+), 129.43 (+), 129.81 (+), 136.31 (q), 142.67 (q), 160.52 (q); Anal. Calc. for C_14_H_18_N_4_O_3_: C, 57.92; H, 6.25; N, 19.30. Found: C, 58.18; H, 6.24; N, 19.32.

*1-((S)-1-Hydroxymethyl-2-phenethyl)-1H-1,2,3-triazole-4-carboxylic acid benzylamide* (**33**): The target compound was obtained by reacting **24** (0.76 mmol, 200 mg) with benzylamine (2.3 mmol, 250 μL) following the procedure described above. The crude product was purified by column chromatography on silica gel using EtOAc/hexane (2:3 v/v) and EtOAc/hexane + 10% MeOH) as eluents to afford **33** as an oil (80 mg, 35% yield). ^1^H-NMR (CDCl_3_) δ 3.22–3.34 (2H, m), 3.99–4.10 (2H, m), 4.56–4.67 (2H, m), 4.76–4.85 (2H, m), 7.08–7.12 (2H, m), 7.21–7.38 (8H, m), 7.46 (1H, t), 8.06 (1H, s); ^13^C-NMR (CDCl_3_) δ 37.75(-), 43.46 (-), 63.71 (-), 65.46 (+), 126.23 (+), 127.51 (*+*), 127.85 (*+*), 128.08 (*+*), 128.97 (*+*), 129.11 (*+*), 136.15 (q), 137.93 (q), 142.66 (q), 160.27 (q); Anal. Calc. for: C_19_H_20_N_4_O_2_: C, 67.84; H, 5.99; N, 16.65. Found: C, 67.68; H, 5.78; N, 16.47.

*1-((R)-1-Hydroxymethyl-2-phenethyl)-1H-1,2,3-triazole-4-carboxylic acid diethylamide* (**34**): The target compound was obtained by reacting 24 (0.69 mmol, 180 mg) with diethyl amine (2.1 mmol, 220 μL) following the procedure described above. The crude product was purified by column chromatography on silica gel using CHCl_3_/EtOAc (2:1 v/v) and, DCM/MeOH (9:1 v/v) as eluents to afford **34** as an oil (25 mg, 12% yield). ^1^H-NMR (CDCl_3_) δ 0.98–1.48 (6H, m), 3.22–3.35 (2H, m), 3.88–3.92 (2H, m), 3.94–4.15 (2H, m), 4.78–4.85 (1H, m), 7.00–7.41 (5H, m), 8.05 (1H, s); ^13^C-NMR (CDCl_3_) δ 12.92 (+), 14.76 (+), 37.61 (-), 41.56 (-), 43.34 (-), 63.61 (-), 65.81 (-), 127.31 (+), 128.12 (+), 128.25 (+), 128.79 (+), 128.91 (+), 129.1 (+), 136.38 (q), 144.51 (q), 160.90 (q); Anal. Calc. for C_16_H_22_N_4_O_2_: C, 63.56; H, 7.33; N, 18.53; Found: C, 63.70; H, 7.36; N, 18.57.

*[1-((S)-1-Benzyl-2-hydroxyethyl)-1H-1,2,3-triazol-4-yl]-morpholin-4-yl-methanone* (**35**): The target compound was obtained by reacting **24** (0.47 mmol, 124 mg) with morpholine (1.4 mmol, 125 μL) following the procedure above. The crude product was purified by column chromatography on silica gel using EtOAc/hexane (3:1v/v) and DCM/MeOH (9;1 v/v) as eluents affording the target compound as an oil (52 mg, 30% yield). ^1^H-NMR (CDCl_3_) δ 3.22–3.33 (2H, m), 3.60–3.85 (4H, d), 3.91–4.04 (2H, m), 4.23–4.31 (4H, d), 4.72–4.83 (1H, m), 7.08–7.09 (2H, m), 7.15–7.26 (3H, m); ^13^C-NMR (CDCl_3_) δ 37.61 (-), 43.22 (-), 47.48 (-), 63.51 (-), 65.34 (+), 66.97 (-), 67.29 (-), 127.34 (+), 128.95 (+), 129.08 (+), 136.28 (q), 143.24 (q), 160.15 (q); Anal. Calc. for: C_16_H_20_N_4_O_3_: C, 60.75; H, 6.37; N, 17.71. Found: C, 60.80; H, 6.39; N, 17.75.

*1-((S)-1-Hydroxymethyl-2-methylpropyl)-1H-1,2,3-triazole-4-carboxylic acid amide* (**36**): The target compound was obtained by reacting **25** (0.49 mmol, 106 mg) with NH_3_ following the procedure above. The crude product was obtained as an oil (32 mg, 35% yield). ^1^H-NMR (CD_3_OD) δ 0.75 (3H, d, *J =* 6.6 Hz), 1.07 (3H, d, *J =* 6.6 Hz ), 2.24–2.37 (1H, m), 3.91–4.12 (2H, m), 4.32–4.44 (1H, m), 8.42 (1H, s); ^13^C-NMR (CD_3_OD) δ 19.31 (+*)*, 19.90 (+*)*, 31.08 (+), 62.83 (-), 71.49(+), 127.51 (+), 133.36 (q), 164.94 (q); Anal. Calc. for C_8_H_14_N_4_O_2_: C, 48.46; H, 7.13; N, 28.27. Found: C, 48.68; H, 7.16; N, 28.35.

*1-((S)-1-Benzyloxymethyl-2-phenethyl)-1H-1,2,3-triazole-4-carboxylic acid amide* (**37**): The target compound was obtained by reacting **27** (0.51 mmol, 180 mg) with NH_3_ following the procedure above. The product was recrystallized from EtOH affording the target compound as a white solid (158 mg, 92% yield). ^1^H-NMR (CDCl_3_) δ 3.20–3.30 (2H, m,, 3.73–3.82 (2H, m), 4.42–4.52 (2H, m), 4.89–4.98 (1H, m), 5.79 (2H, s), 6.99–7.10 (2H, m), 7.16–7.36 (8H, m), 8.11 (1H, s); ^13^C-NMR (CDCl_3_) δ 37.98 (-), 63.54 (+), 70.22 (-), 73.72 (-), 126.24 (+), 127.44 (+), 128.01 (+), 128.27(+), 128.77 (+), 129.00 (+), 129.09 (+), 136.0 (q), 137.2 4(q), 142.34 (q), 162.31 (q); Anal. Calc. for: C_19_H_20_N_4_O_2_: C, 67.84; H, 5.99; N, 16.55. Found: C, 67.73; H, 5.93; N,16.49.

*1-((S)-1-Benzyloxymethyl-2-phenethyl)-1H-1,2,3-triazole-4-carboxylic acid butylamide* (**38**): The target compound was obtained by reacting **27** (0.56 mmol 200 mg) with butyl amine (1.7 mmol, 124 mg) following the procedure above. The target compound was obtained as an oil (207 mg, 93% yield) without any purification. ^1^H-NMR (CDCl_3_) δ 0.92–0.98 (3H, m), 1.37–1.45 (2H, m), 1.55–1.65 (2H, m), 3.19–3.33 (2H, m), 3.40–3.48 (2H, m), 3.72–3.83 (2H, m), 4.41–4.53 (2H, dd), 4.89–4.99 (1H, m), 7.01–7.07 (2H, m), 7.17–7.37 (8H, m), 8.12 (1H, s); ^13^C-NMR (CDCl_3_) δ 13.87 (+*)*, 20.19 (-), 31.78 (-), 37.87 (-), 38.94 (-), 63.33 (+), 70.22 (-), 73.56 (-), 125.51 (+), 127.28 (+), 127.89 (+), 128.12 (+), 128.64 (+), 128.87 (+), 128.99 (+), 136.05 (q), 137.21 (q), 143.10 (q), 160.24 (q); Anal. Calc. for: C_23_H_28_N_4_O_2_: C, 70.38; H, 7.19; N, 14.27; Found: C, 70.34; H, 7.24; N, 14.36; *t*_R_ (Method B): 21.2.

*1-((S)-1-Benzyloxymethyl-2-phenethyl)-1H-1,2,3-triazole-4-carboxylic acid (2-hydroxyethyl)-amide* (**39**): The target compound was obtained by reacting **27** (0.56 mmol, 200 mg) with ethanol amine (1.7 mmol, 104 mg) following the procedure above. The target compound was obtained as an oil (170 mg, 81% yield) without any purification. ^1^H-NMR (CDCl_3_) δ 3.16–3.30 (2H, m), 3.55–3.61 (2H, m), 3.71–3.80 (4H, m), 4.39–4.50 (2H, m), 4.88–4.97 (1H, m), 7.00–7.04 (2H, m), 7.14–7.34 (8H, m), 7.87 (1H, m), 8.18 (1H, s); ^13^C-NMR (CDCl_3_) δ 37.77 (-) , 42.18 (-) , 61.88 (-), 63.31 (+), 70.19 (-), 73.47 (-), 125.86 (+), 127.23 (+), 127.84 (+), 128.08 (+), 128.59 (+), 128.82 (+), 128.94 (+), 135.97 (q), 137.17 (q), 142.72 (q), 161.1 (q); Anal. Calc. for: C_21_H_24_N_4_O_3_: C, 65.38; H, 6.31; N, 15.25; Found: C, 65.45; H, 6.29; N, 15.23.

*1-((S)-1-Benzyloxymethyl-2-phenethyl)-1H-1,2,3-triazole-4-carboxylic acid benzylamide* (**40**): The target compound was obtained by reacting **27** (0.42 mmol, 150 mg) with benzyl amine (1.2 mmol, 137 mg) following the procedure above. The target compound was obtained as an oil (161 mg, 87% yield) without any purification. ^1^H-NMR (CDCl_3_) δ 3.19–3.33 (2H, m), 3.73–3.82 (2H, m), 4.42–4.53 (2H, m), 4.56 (2H, d, *J =* 5.9 Hz), 4.89–4.98 (1H, m), 7.02–7.07 (2H, m), 7.18–7.38 (13H, m), 7.49 (1h, t, *J =* 5.9Hz), 8.12 (1H, s); ^13^C-NMR (CDCl_3_) δ 37.96 (-), 43.31 (-), 63.47 (+), 70.26 (-), 73.70 (+), 125.80 (+), 127.40 (+), 127.73 (+), 128.00 (+), 128.06 (+), 128.24 (+), 128.75 (+), 128.90 (+), 128.98 (+), 129.08 (+), 136.09 (q), 137.26 (q), 138.12 (q), 142.90 (q), 160.24 (q); Anal. Calc. for C_26_H_25_N_4_O_2_: C: 73.22; H, 6.14; N, 13.14. Found: C, 73.28; H, 6.12; N, 13.19.

*1-((S)-1-Benzyloxymethyl-2-phenethyl)-1H-1,2,3-triazole-4-carboxylic acid diethylamide* (**41**): The target compound was obtained by reacting **27** (0.56 mmol, 200 mg) with diethylamine (1.7 mmol, 125 mg) following the procedure above. The crude product was purified by column chromatography on silica gel (hexane, then 1:1 EtOAc/hexane) to afford the desired amide as an oil (17 mg, 8% yield). ^1^H- NMR (CDCl_3_) δ 1.18–1.35 (6H, m), 3.20–3.36 (2H, m), 3.46–3.58 (2H, m), 3.70–3.98 (4H, m), 4.42–4.55 (2H, m), 4.87–4.97 (1H, m), 7.00–7.39 (10H, m, *Ph*), 8.09 (1H, s, C*CH*N); ^13^C-NMR (CDCl_3_) δ 13.04 (+), 14.81 (+), 38.08 (-), 41.35 (-), 43.27 (-), 63.14 (+), 70.34 (-), 73.69 (-), 127.33 (+), 127.9 (+), 128.1 (+), 128.2 (+), 128.7 (+), 129.1 (+), 136.3 (q), 137.18 (q), 144.5(q), 160.9 (q); Anal. Calc. for C_23_H_28_N_4_O_2_: C, 70.38; H, 7.19; N, 14.27. Found: C, 70.40; H, 7.16; N, 14.28.

*[1-((S)-1-Benzyloxymethyl-2-phenyethyl)-1H-1,2,3-triazol-4-yl]-morpholin- 4-yl-methanone* (**42**): The target compound was obtained by reacting **27** (0.56 mmol, 200 mg) with morpholine (1.7 mmol, 148 mg) following the procedure above. The target compound was obtained as an oil (182 mg, 82% yield) without any purification. ^1^H-NMR (CDCl_3_) δ 3.18–3.32 (2H, m), 3.71–3.78 (8H, m), 4.27–4.33 (2H, m), 4.36–4.49 (2H, m), 4.88–4.96 (1H, m), 6.97–7.32 (10H, m), 8.13 (1H, s); ^13^C-NMR (CDCl_3_) δ 37.84 (-), 43.07 (-), 47.35 (-), 63.14 (+), 66.95 (-), 67.28 (-), 70.16 (-), 73.52 (-), 127.25 (+), 127.83 (+), 128.08 (+), 128.61 (+), 128.67 (+),128.82 (+), 128.99 (+), 136.07 (q), 137.23 (q), 143.65 (q), 160.01 (q); Anal. Calc. for C_22_H_23_N_4_O_3_: C, 67.96; H, 6.45; N, 13.78. Found: C, 68.04; H, 6.41; N, 13.72.

*1-((R)-1-Benzyloxymethyl-2-phenethyl)-1H-1,2,3-triazole-4-carboxylic acid butylamide* (**43**): The target compound was obtained by reacting **28** (0.56 mmol, 200 mg) with butyl amine (1.7 mmol, 124 mg) following the procedure above. The target compound was obtained as an oil (220 mg, 98% yield) without any purification. ^1^H-NMR (CDCl_3_) δ, 0.96 (3H, t, *J* = 7.3Hz), 1.37–1.47 (2H, m), 1.56–1.65 (2H, m), 3.19–3.34 (2H, m), 3.41–3.47 (2H, m), 3.72–3.83 (2H, m), 4.42–4.57 (2H, dd, *J =* 11.9 Hz, *J =* 25.5Hz), 4.90–4.98 (1H, m), 7.03–7.07 (2 H, m), 7.17–7.38 (8H, m), 8.13 (1H, s); ^13^C-NMR (CDCl_3_) δ 13.88 (+*)*, 20.21 (-), 31.79 (-), 37.88 (-), 38.96 (-), 63.34 (+), 70.25 (-), 73.59 (-), 125.53 (+), 127.30 (+), 127.90 (+), 128.12 (+), 128.66 (+), 128.89 (+), 129.01 (+), 136.06 (q), 137.22 (q), 143.10 (q), 160.25 (q); Anal. Calc. for C_23_H_28_N_4_O_2_: C, 70.38; H, 7.19; N, 14.27. Found: C, 70.46; H, 7.16; N, 14.21; *t*_R_ (Method B): 14.9.

*1-((S)-1-Benzyloxymethyl-3-phenylpropyl)-1H-1,2,3-triazole-4-carboxylic acid amide* (**44**): The target compound was obtained by reacting **29** (0.15 mmol, 57 mg) with NH_3_ following the procedure above. The crude product was purified by column chromatography using EtOH/hexane (1:1 v/v) as eluent. The target compound was obtained as a white solid (44 mg, 88% yield). ^1^H-NMR (CDCl_3_) δ 2.15–2.28 (1H, m), 2.33–2.57 (3H, m), 3.68–3.79 (2H, m), 4.38–4.52 (2H, dd, *J* = 12.1 Hz, *J =* 30.0 Hz), 4.67–4.75 (1H, m), 7.05–7.34 (10H, m), 8.22 (1H, s); ^13^C-NMR (CDCl_3_) δ 31.90 (-), 33.08 (-), 61.41 (+), 71.39 (-), 73.63 (-), 126.04 (+), 126.64 (+), 127.93 (+), 128.24 (+), 128.59 (+), 128.75 (+), 128.85 (+), 137.27 (q), 140.01 (q), 142.65 (q), 162.47 (q); Anal. Calc. for C_20_H_22_N_4_O_2_: C, 68.55; H, 6.33; N, 15.99. Found: C, 68.48; H, 6.26; N, 15.88.

*1-((S)-1-Benzyloxymethyl-3-phenylpropyl)-1H-1,2,3-triazole-4-carboxylic acid butylamide* (**45**): The target compound was obtained by reacting **29** (0.3 mmol, 113 mg) with butylamine (0.9 mmol, 67 mg) following the procedure above. The target compound was obtained as an oil (113 mg, 93% yield) without any purification. ^1^H-NMR (CDCl_3_) δ 0.95 (3H, t, *J =* 7.3 Hz), 1.37–1.47 (2H, m), 1.56–1.67 (2H, m), 2.15–2.56 (4H, m), 3.44–3.49 (2H, m), 3.67–3.77 (2H, m), 4.43 (2H, dd, *J =* 12.1 Hz, *J =* 29.3 Hz), 4.65–4.73 (1H, m), 7.04–7.35 (10H, m), 8.18 (1H, s); ^13^C-NMR (CDCl_3_) δ 13.89 (*+*), 20.22 (-), 31.82 (-), 31.82 (-), 33.03 (-), 38.98 (-), 61.24 (-), 71.39 (-), 73.52 (-),125.30 (+), 126.52 (+), 127.82 (+),128.11 (+), 128.51 (+), 128.64 (+), 128.75 (+), 137.24 (q), 140.01 (q), 143.33 (q), 160.30 (q); Anal. Calc. for C_24_H_30_N_4_O_2_: C, 70.91; H, 7.44; N, 13.78. Found: C, 71.05; H, 7.39; N, 13.86.

*1-[(S)-1-Benzyloxymethyl-2-(tert-butyl-dimethylsilanyloxy)-ethyl]-1H-1,2,3-triazole-4-carboxylic acid amide* (**46**): The target compound was obtained by reacting **30** (0.23 mmol, 95 mg) with NH_3_ following the procedure above. The product was recrystallized from EtOH affording **46** as a white solid (88 mg, 92% yields). ^1^H-NMR (CDCl_3_) δ -0.09(3H, s), -0.01 (3H, s), 0.82 (9H, s), 3.81–4.03 (5H, m), 4.41–4.52 (2H, m), 4.81–4.87 (1H, m), 7.11–7.25 (5H, m), 8.16 (1H, s); ^13^C-NMR (CDCl_3_) δ -5.48 (*+*), -5.42 (+), 18.31 (q), 62.10 (-), 62.64 (+), 67.74 (-), 73.24 (-), 125.56 (+), 127.79 (+), 128.31 (+), 128.73 (+), 137.01 (q), 142.23 (q), 161.03 (q); Anal. Calc. for C_19_H_30_N_4_O_3_Si: C, 58.43; H, 7.74; N, 14.35. Found: C, 58.58; H, 7.79; N, 14.42.

### 3.7. Docking

X-ray structures with inhibitors were used as starting point for all dockings. The enzymes were prepared according to the standard procedure in the Schrödinger package. Docking was performed by using Glide (Schrödinger) [37,38,39] with extra precision (XP) settings and standard parameters for ligand docking.

## 4. Conclusions

An efficient synthetic route was developed for the synthesis of chiral 1,4-disubstituted-1,2,3-triazole derivatives starting from D-or L-amino acids. The target compounds were obtained in excellent yields with no racemization. This straight forward and flexible strategy allow the synthesis of novel, 4-disubstituted-1,2,3-triazole derivatives with desired properties and is currently being explored in our laboratory for the development of kinase inhibitors.
